# The administration of high-dose propofol sedation with manual and target-controlled infusion in children undergoing radiation therapy: a 7-year clinical investigation

**DOI:** 10.1186/s40064-016-2011-1

**Published:** 2016-03-25

**Authors:** Tak Kyu Oh, Seung Jae Lee, Jae Hyun Kim, Boram Park, Woosik Eom

**Affiliations:** Department of Anesthesiology and Pain Medicine, National Cancer Center, 323, Ilsan-ro, Ilsandong-gu, Goyang-si, Gyeonggi-do 10408 Republic of Korea; Biometric Research Branch, Research Institute and Hospital, National Cancer Center, 323, Ilsan-ro, Ilsandong-gu, Goyang-si, Gyeonggi-do 10408 Republic of Korea

**Keywords:** Propofol, Pediatrics, Proton therapy, Manual infusion, Target-controlled infusion

## Abstract

**Background:**

Radiation therapy requires the patient to remain immobile for a long time, which is challenging in children. This study therefore aimed to determine the adequate target concentration and dosage of propofol in target-controlled infusion (TCI) and manual infusion (MI) in children requiring sedation for proton radiation therapy. Our hypothesis is that the adequate dose of propofol sedation required for proton radiation therapy in pediatric patients was larger than that seen in previous studies.

**Methods:**

We retrospectively analyzed the medical records of Korean children who received proton therapy under propofol sedation. The average target concentration at induction and during maintenance with TCI and the dose with MI were analyzed as primary outcomes.

**Results:**

A total of 1296 procedures in 54 children were analyzed (TCI group, 26; MI group, 28). The median bolus dose of propofol in the MI group was 2.6 (2.2–3.0) mg/kg, while the pump speed was 17.0 (13.6–25.8) mg/kg/h. The median target concentration of propofol in the TCI group was 5.3 (4.4–5.7) mcg/mL at induction and 4.2 (3.1–5.1) mcg/mL during maintenance. There were no cases of life-threatening complications in either group over 7 years. There were six cases of transient desaturation, which were managed by using the jaw thrust maneuver.

**Conclusions:**

Compared with those in previous studies, the target concentration of propofol with TCI and the propofol dose with MI required for adequate sedation in children undergoing proton radiation therapy were larger in the present study. Despite concerns regarding overdosage, the complications were managed well. However, safe and adequate sedation for proton radiation therapy remains a challenge. The development of monitoring tools to evaluate the depth of sedation is necessary to adjust the propofol dose and sedation level.

## Background

Effective sedation in children is accompanied by problems related to maintenance of the patient’s airway, drug selection, and drug dose. A long period of immobility is required during magnetic resonance imaging (MRI) or radiation therapy, and in children, this can be achieved only with sedation. Because the demand for procedural sedation of children outside the operating room is continuously increasing, effective and safe sedation has become an important issue for anesthesiologists and other medical professionals (Gozal and Gozal 2008). Propofol alone is sufficient for effective sedation in pediatric patients undergoing painless procedures such as radiation therapy (Cravero and Blike 2004).

Proton therapy, which destroys abnormal tissues in the body via delicate control of the range of a proton beam, requires deep sedation in children to avoid movement and maintain a precise position during irradiation. Proton therapy facilities are usually located far away from central operating rooms, and it is generally performed in pediatric outpatients or patients with cerebrospinal tumours; therefore, a precise and harmless method of sedation is desirable (Frei-Welte et al. 2012).

Efforts to establish propofol infusion regimens for deep sedation in pediatric patients undergoing radiation therapy have been made. Scheiber et al. (1996) reported that a propofol loading dose of 3.6 ± 0.59 mg/kg that was immediately followed by continuous infusion of 7.4 ± 2.2 mg/kg/h for maintenance was adequate and safe for deep sedation in children undergoing radiation therapy. Buehrer et al. (2007) reported that a propofol loading dose of 3.7 mg/kg that was immediately followed by continuous infusion of 10 mg/kg/h did not cause problems in children undergoing proton therapy. We have performed pediatric sedation with manual infusion (MI) or target-controlled infusion (TCI) for a long time, and the desired level of sedation has been similar with both methods. Therefore, comparison of the two regimens can provide clinical evidence for propofol infusion in pediatric patients and clues regarding a compatible dose for TCI and MI.

The aim of the present study was to determine the adequate target concentration and dosage of propofol in TCI and MI in children requiring sedation for proton radiation therapy. Our hypothesis was that the adequate dose of propofol sedation for proton radiation therapy in pediatric patients was larger than that seen in previous studies.

## Methods

This retrospective observational study included pediatric patients who received proton therapy under propofol sedation according to the same protocol at the National Cancer Center from April 2007 to November 2013. Data were obtained from the electronic medical records of patients. To avoid reporting bias, an independent investigator collected electronic medical records. This study was conducted with the approval of the Institutional Review Board for Health Science Research of the National Cancer Center of Korea (IRB No., NCC2014-0026).

Pediatric sedation using propofol was performed according to an established protocol at the Department of Anesthesiology at the National Cancer Centre. From April 2007 to September 2011, anesthesiologists performed sedation by controlling the rate of propofol infusion per hour on the basis of the patient’s condition using a conventional infusion pump (Pilot C, Fresenius Vial S.A., Brézins, France). From October 2011 to November 2013, sedation was performed with a TCI pump (Syramed uSP 6000, Acromed, Regensdorf, Switzerland) that was capable of TCI with the Paedfusor model, which is pharmacokinetically applicable to pediatric patients aged 1–15 years (Absalom et al. 2003). A total of four anesthesiologists, including the Director of pediatric sedation for proton radiation therapy (W.E.), sedated the patients using the same protocol.

### Sedation protocol

One week before starting actual radiation therapy, all the pediatric patients underwent simulations to understand radiation therapy and to determine the exact individual regimen they would need for propofol sedation. In simulation sessions, all the pediatric patients undergo a test to adjust the adequate dose of propofol for proton radiation therapy. Without any premedication, the anesthesiologists initiated simulation with a low dose of propofol (2–3 mg/kg bolus followed 10 mg/kg/h of continuos infusion in the MI group and 3 mcg/mL of the target concentration in the TCI group). The anesthesiologists elevated the dose of propofol gradually (1–2 mg/kg/h in the MI group and 0.2–0.3 mcg/mL in the TCI group) to maintain immobility for positioning with a tight-fitting mask. If the simulation for proton radiation therapy was safely initiated without any movement, the anesthesiologist considered the dose to be adequate for sedation. The entire process of simulation was cautiously performed by anesthesiologists with routine monitoring (noninvasive blood pressure monitoring at 5-min intervals, oxyhemoglobin saturation monitoring, electrocardiography, and capnometry).

After individual target concentration of sedation for TCI, or the infusion regimen for MI was determined in simulation sessions, scheduled proton radiation treatments were performed for 2–5 weeks, daily except Saturday and Sunday; each patient underwent a total of 20–30 sedations.

The patients were not premedicated. All patients were subjected to noninvasive blood pressure monitoring at 5-min intervals, oxyhaemoglobin saturation monitoring, electrocardiography, and capnometry. All data were collected automatically by using the electronic medical record system. In addition, special events in sedation, if any, were recorded directly by anesthesiologists. Endotracheal intubation and general anesthesia were made available in case of emergency. Oxygen was supplied at 3 L/min through an oxygen mask that was placed over the face-fitting mask during sedation, and ventilation was confirmed with exhalation of carbon dioxide in both groups by capnometry (Vamos, Drager™, Lubeck, Germany); a line for detection carbon dioxide was placed in the oxygen mask.

Propofol infusion was discontinued after proton therapy, and patients were moved to the postanesthesia care unit (PACU), placed 10 m away from the radiation therapy room. Oxygen was supplied at 3 L/min through an oxygen mask with oxyhaemoglobin saturation monitoring when moving the patient to the PACU.

### Infusion regimen for sedation

In the MI group, sedation was induced by injecting 2–3 mg/kg of 1 % propofol according to the patient’s age; 1 % propofol was infused using a conventional MI pump. Because a radiation chamber is not accessible during proton beam irradiation, sedation was maintained by stepping down the propofol infusion rate by 1–2 mg/kg/h at irregular intervals between beams. The initial infusion rate was determined in the first simulation session, the data of which are excluded when calculating the result of this study.

For sedation in the TCI group, 2 % propofol was administered at the target concentration predetermined during the simulation session. The target concentration for induction was defined as the highest target concentration for positioning and tight mask fitting in the TCI group during the induction period. After induction, the target concentration was decreased by 0.5–1.5 mcg/mL to the lowest level considered adequate for the maintenance of sedation by the anesthesiologists; this lowest level was defined as the target concentration for maintenance. The initial target concentration was determined in the simulation session for this procedure as well.

### Other clinical variables

The average daily propofol dose for sedation during each session was calculated by dividing the total propofol dose (mcg) (the sum of the amount used in all 1296 sessions) by the weight of the patient (kg) and the sedation time (min). The recovery time was recorded accurately as the time (min) from arrival in the recovery room to discharge at the discretion of the attending physician. According to the modified Aldrete scoring system, an appropriate state of consciousness, smooth breathing, normal cardiovascular vital signs, and normal movement were used as discharge criteria.

To assess the incidences of adverse events (AEs), all desaturation events were included, regardless of duration, if the oxygen saturation decreased below 90 %. Bradycardia and hypotension were counted if the heart rate or mean blood pressure dropped by more than 25 % relative to the initial values. In addition, data regarding the use of vasopressors, anticholinergics, nasopharyngeal airway device, jaw thrust maneuver, and mask ventilation for the management of AEs were collected from the medical records to evaluate the clinical significance of AEs.

The primary outcomes of this study were the target concentration of propofol with TCI and propofol dose with MI for pediatric sedation during proton radiation therapy. The secondary outcomes were the average daily propofol dose, recovery time, and incidences of AEs in both groups.

### Statistical analysis

The patient characteristics are summarized in Table 1, with numbers and percentages for categorical variables and medians (interquartile ranges) for continuous variables. Pearson’s Chi square test or Fisher’s exact test was used for comparisons of categorical variables between groups, while the Wilcoxon rank-sum test was used for comparison of continuous variables. The incidences of AEs were represented as numbers and percentages. All statistical analyses were performed using SAS (version 9.3; SAS Institute, Cary, NC, USA) and R (version 3.02) software.

**Table 1:** Patient characteristics

Characteristic	Target-controlled infusion	Infusion pump	*p* value
	(N = 26)	(N = 28)	
Sex [n (%)]			
Female	13 (50.0)	14 (50.0)	1.000
Male	13 (50.0)	14 (50.0)	
Age			
Median (IQR)	4 (3–5)	3 (2–5)	0.169
Weight (kg)			
Median (IQR)	16.3 (12–20)	13.8 (11.3–18.0)	0.199
Session			
Median (IQR)	23.5 (17–30)	24.5 (20.5–27.5)	0.775
Sedation time (min)			
Median (IQR)	57.8 (53.0–65.0)	44.2 (36.8–53.6)	<0.001^†^
Recovery time (min)			
Median (IQR)	52.2 (46.9–57.0)	33.3 (26.1–40.3)	<0.001^†^
Prone position [n (%)]			
No	25 (96.2)	25 (89.3)	0.612
Yes	1 (3.8)	3 (10.7)	
Lesion [n (%)]			
Brain^a^	5 (19.2)	18 (64.3)	<0.001^†^
Brain and spinal cord	15 (57.7)	2 (7.1)	
Others^b^	6 (23.1)	8 (28.6)	

*IQR* interquartile range

^†^Wilcoxon rank-sum test

^a^Brain, brain stem

^b^Head and neck, pelvis, spine

## Results

This retrospective observational study included a total of 1296 procedures and 54 pediatric patients who received proton therapy under propofol sedation with TCI (n = 26) or MI (n = 28). There were no statistically significant differences between the two groups with regard to demographic and clinical characteristics, including sex, age, weight, and use of the prone position, while significant differences were detected in sedation time, recovery time, and target lesion (Table 1). The sedation time was significantly greater in the TCI group (57.8 min) than in the MI group (44.2 min; p < 0.001), as was the average recovery time (52.2 min vs. 33.3 min; p < 0.001). The proportion of patients with both brain and spinal cord lesions was significantly higher in the TCI group (57.7 %) than in the MI group (7.1 %; p < 0.001).

The median bolus dose of propofol in the MI group was 2.6 mg/kg (2.2–3.0), while the pump speed was 17.0 (13.6–25.8) mg/kg/h. The median target concentration of propofol was 5.3 (4.4–5.7) mcg/mL at induction and 4.2 (3.1–5.1) mcg/mL during maintenance in the TCI group (Table 2). The average propofol dose was not significantly different between groups (TCI: 301.5 mcg/kg/min; MI: 254.6 mcg/kg/min; p = 0.149; Table 2).

**Table 2:** Propofol-related parameters

Parameter	Target-controlled infusion	Infusion pump	*p* value
	(N = 26)	(N = 28)	
Target concentration (induction, mcg/mL)	5.3 (4.4–5.7)	–	
Target concentration (maintenance, mcg/mL)	4.2 (3.1–5.1)	–	
Induction dose (mg/kg)	–	2.6 (2.2–3.0)	
Maintenance dose (mg/kg/h)	–	17.0 (13.6–25.8)	
Average dose (mcg/kg/min)	301.5 (235.4–354.0)	254.6 (215.6–299.1)	0.149

Data are presented as median (interquartile range)

A >25 % decrease in the initial heart rate was observed in 10 of the 54 patients. Six of these received anticholinergics at the discretion of the anesthesiologists. A >25 % decrease in the mean blood pressure was recorded in only one of the 54 patients. No patient required vasopressors, and no patient exhibited a >50 % decrease in the heart rate or mean blood pressure. Six of the 54 patients (11 %) experienced desaturation below 90 % during the induction period. However, they all recovered immediately after the jaw thrust maneuver was performed by the anesthesiologist. No patient required mask ventilation or endotracheal intubation. Two patients experienced sinus arrhythmia that recovered after sedation, and one experienced urticaria necessitating antibiotic infusion during sedation (Table 3).

**Table 3:** Adverse events

Adverse event [n (%)]	Target-controlled infusion	Infusion pump
	(N = 26)	(N = 28)
None	20 (71.4)	21 (63.6)
Bradycardia	4 (14.3)	6 (18.2)
Hypotension	0 (0.0)	1 (3.0)
Desaturation	3 (10.7)	3 (9.1)
Others^a^	1 (3.6)	2 (6.1)

^a^Arrythmia, urticaria

## Discussion

In this study, pediatric patients receiving proton radiation therapy required a larger dose of propofol for sedation compared with patients in previous studies (Scheiber et al. 1996; Buehrer et al. 2007). Several factors should be considered when comparing the present study with previous studies. First, the therapeutic range of propofol is wider than that seen in earlier studies. In a study (Jayabose et al. 2001), a median value of 25.0 mg/kg/h (range 3.0–94.2) of propofol was used for sedating patients undergoing several procedures (e.g., lumbar puncture, bone marrow aspiration) in patients ages 0–2 years without any life-threatening adverse event. Although the sedation was not performed for radiation therapy in this study, we noted that the therapeutic range was wider than what we expected for propofol sedation in pediatric patients.

Second, in the MI group, our induction bolus dose was smaller than that seen in three earlier studies (Scheiber et al. 1996; Buehrer et al. 2007; Weiss et al. 2007). We used a median value of 2.6 mg/kg (IQR 2.2–3.0) as an induction dose. Considering the sedation time (usually <1 h), our maintenance dose could be larger than that needed in other studies to maintain immobility during the procedure. Considering the anxiety levels of children and patients, we needed to apply higher target concentrations: 5.3 (4.4–5.7) mcg/ml for induction and 4.2 (3.1–5.1) mcg/ml for maintenance. However, to the best of our knowledge, there are no previous reports on pediatric sedation for radiation therapy using TCI, with which to compare our study.

Third, in a study by Buehrer et al. (2007), 0.1 mg/kg midazolam was used before induction, which may have affected the propofol dose required for deep sedation. In another study by Jayabose et al. (2001), additional use of benzodiazepine with propofol infusion was seen to reduce the requirements of propofol by about 25 % in children.

Considering the increased dose used for sedation, a major concern in our study was AEs. The incidence of cardiovascular depression was considered insignificant because the ≥25 % decrease in heart rate or blood pressure was easily recovered by decreasing the infusion speed or administering anticholinergics. Desaturation occurred during the induction period in six patients; however, all of them easily recovered with the jaw thrust maneuver. The incidence of AEs was higher in our study than in the study by Weiss et al. (2007) that reported no respiratory adverse event. However, we analyzed 1296 procedures, and only six cases occurred—one desaturation event in each of six patients who recovered easily after the jaw-thrust maneuver. It was a lower percentage of incidence than we estimated, and we believe that several factors made this possible.

First, the main AE was more likely to occur with the induction bolus dose. A study by Dundee et al. (1986) demonstrated that the dose of induction and rapid speed of injection in the induction period when using propofol are the main factors in the occurrence of AEs like apnea and hypotension; however, this study had been conducted in elderly patients. Our study used a smaller induction dose than seen in previous studies, which reduced the possible harmful effects of the sedation (Scheiber et al. 1996; Buehrer et al. 2007; Weiss et al. 2007). Second, the simulations to find the adequate dose or target concentration for safe sedation were performed carefully before the actual sedations. Third, professional anesthesiologists closely monitored the patients from a monitoring room by using video monitors during sedation. Fourth, as reported by Jayabose et al. (2001), the therapeutic range for safe sedation was wider than we had initially anticipated.

This study included not only MI, but also TCI, making it different from previous studies (Scheiber et al. 1996; Buehrer et al. 2007). Based on our experience, we believe TCI has two advantages. First, the anesthesiologists were not required to intervene frequently to maintain adequate sedation, because in TCI, the infusion speed changed automatically to reach the target concentration in brain. Second, the initial bolus dose through intravenous injection was not needed in the induction period, making the induction easier for anesthesiologists to perform. However, several TCI models are available for use in younger patients, which can lead to different results among models (Sepulveda et al. 2011). Therefore, further studies are necessary to determine the safest and most efficient use of propofol TCI for pediatric sedation.

This study has some limitations. First was its retrospective, single-center design with data collection over a long period. Second, all physicians used the same protocol for sedation, although judgments of the sedation level and dose may have been affected by the physician’s experience. Third, the two methods were not used in the same period; therefore, the experiences acquired during MI may have influenced sedation with TCI. Fourth, as endotracheal intubation was not performed, the respiration rate and end tidal carbon dioxide were not accurately measured as a respiratory parameter.

Nevertheless, to the best of our knowledge, this is the first study to analyze both the target propofol concentration with TCI and the propofol dose with MI for pediatric sedation during radiation therapy.

## Conclusions

Compared with those in previous studies, the target concentration of propofol with TCI and the propofol dose with MI required for adequate sedation in children undergoing proton radiation therapy were larger in the present study. Despite concerns of overdosage, the AEs in the present study were easily managed by anesthesiologists. However, safe and adequate sedation for proton radiation therapy remains a challenge. The development of monitoring tools to evaluate the depth of sedation is necessary to adjust the dose of propofol and the level of sedation. Further studies in the field of imaging and radiotherapy are necessary to discover better alternatives involving a lower dose of propofol and a decreased incidence of adverse events in pediatric patients requiring sedation for proton therapy.
